# Traditional Milk Marketing Chain: One of the Leading Causes of Fatal Diseases in Pakistan

**Published:** 2018-07

**Authors:** Anam KHURSHID, Ahmed USMAN, Umer FAROOQ

**Affiliations:** 1. Dept. of Public Health, Institute of Social and Cultural Studies, University of the Punjab, Lahore, Pakistan; 2. Dept. of Sociology, Institute of Social and Cultural Studies, University of the Punjab, Lahore, Pakistan; 3. University College of Veterinary and Animal Sciences, The Islamia University of Bahawalpur, Punjab, Pakistan

## Dear Editor-in-Chief

Dairy sector in Pakistan plays an important role in national economy with annual milk production of about 33 million tonnes, making it fourth largest milk producing country in the world (1). Despite this, Pakistan is facing a number of problems in quality milk production. Lack of dairy-related education, commercial dairy farming, presence of middleman milk system, lack of financial and infrastructure facilities and absence of appropriate milk adulteration test system at field level (2) result in various health problems such as cholera, skin diseases, hepatitis, blood pressure, and kidney disorders.

In Pakistan, the majority of small dairy holders are laymen. They strictly follow traditional practices of milk marketing. Since milk is purchased on the basis of milk fat and solids-not-fat percentage, the middlemen practice adulteration in order to attain maximum level of these parameters. Apart from pond or canal water adulteration, many other dangerous ingredients such as detergents, formalin, vegetable oils, hair removing powder, urea, and sugar mixture in milk have also been reported in Pakistan (3). The analysis of commercial packaged Ultra High Temperature (UHT) and pasteurized milk has revealed that many of giant companies of milk industry are also selling a “hub of diseases” in the name of milk. Adulteration of milk causes significant human health problems including gastric ulcers, diarrhea, colon ulcers, loss of acquired immunity, vomiting, abdominal pain, metabolic acidosis, and urinary tract infections in infants (3). Organizational infrastructure of milk marketing chain is found to be insufficient to ensure a quality product. Proper transportation of milk also requires an interconnected cold chain to maintain its quality (4). Raw milk is the primary dairy product which is being marketed in the country (5). So implementation of proper milk analysis system is necessary to cope with this problem. Livestock and Dairy Development Department of Punjab Province have recently taken certain initiatives to establish basic milk quality assurance test system.

Both at rural and commercial levels, proper registration of small dairy farmers are required. Establishment of trained quality assurance teams is a dire need of time in order to supervise the quality and nutritious value of milk by analyzing samples on weekly or monthly basis for both commercially packaged milk and rural farmers. Strict Policies should be made to stop non-registered farmers from selling milk. Instead of allowing private milk companies to grow their business, government should establish some monitoring and evaluation system for quality and adulteration control on regular basis. Achieving our goal of providing pure milk to the consumer is only possible through extension services to the farmers, awareness of consumer, strict monitoring and implementation of milk quality test system. Research on production structure in dairying can enable to understand structural changes needed in dairy sector (6).
